# Shaping the battlefield: *EGFR* and* KRAS* tumor mutations’ role on the immune microenvironment and immunotherapy responses in lung cancer

**DOI:** 10.1007/s10555-025-10272-4

**Published:** 2025-06-17

**Authors:** Bassel Alsaed, Nina Bobik, Hanna Laitinen, Tanvi Nandikonda, Ilkka Ilonen, Heidi M. Haikala

**Affiliations:** 1 Translational Immunology Research Program (TRIMM), Research Programs Unit, Faculty of Medicine, University of Helsinki, Helsinki, Finland; 2 iCAN Digital Precision Cancer Medicine Flagship, University of Helsinki, Helsinki, Finland; 3 Department of General Thoracic and Esophageal Surgery, Heart and Lung Center, Helsinki University Hospital & University of Helsinki, Helsinki, Finland; 4 Faculty of Medicine and Health Technology, Tampere University, Tampere, Finland

**Keywords:** Lung cancer, Immunotherapy, Tumor microenvironment, *EGFR*, *KRAS*

## Abstract

The two most common and mutually exclusive driver mutations in non-small cell lung cancer affect *EGFR* and *KRAS* oncogenes. While *EGFR* mutations typically arise in non-smokers and are correlated with non-inflamed tumor microenvironment, *KRAS* mutations are associated with tobacco smoking, high mutational burden, and immunologically more active tumors. Consequently, current cancer immunotherapies have failed in patients with *EGFR* mutations, while patients with *KRAS* mutations have more favorable outcomes. The distinctive properties of the tumor immune microenvironment can partly explain the differences in the treatment outcomes. Besides the undeniable role of T lymphocytes, other immune cell types, cancer-associated fibroblasts, immunomodulatory cytokines, and angiogenesis are emerging as important players in these tumors. This article summarizes the current knowledge about the impact of *EGFR* versus *KRAS* mutations, among other mutations, on the tumor microenvironment and immunotherapy responses in lung cancer, highlighting the possible clinical implications for present and upcoming immunotherapy regiments, as well as emphasizing the gaps in the current knowledge that should be further investigated.

## Introduction

Non-small cell lung cancers (NSCLCs) account for approximately 85% of all lung cancers, with lung adenocarcinomas as the most predominant histological subtype. *EGFR* and *KRAS* are well-known oncogenes established as the most common driver mutations within these tumors [1]. The two mutations are mutually exclusive and show distinct incidence in relation to the ethnicity and smoking history of the patients. Furthermore, their differential responsiveness to currently available immunotherapies underscore the importance of personalized treatment choices [2]. The underlying factors contributing to the immunotherapy responses in *EGFR*- and *KRAS*-mutated NSCLCs remain uncertain. Still, these effects are believed to be mediated by specific components within the tumor (immune) microenvironment (TME/TIME). This review outlines the current understanding of how *EGFR*, *KRAS,* and other key mutations influence immunotherapy responses and tumor immunology. Additionally, recent studies investigating the composition of the TME/TIME in *EGFR*- versus *KRAS*-mutated NSCLC are summarized. By better understanding the intricacies of the TME/TIME in these prevalent mutation types, we could further advance novel therapies targeting not only the tumor cells but also their malignant TME/TIME.

### EGFR in lung cancer

**Fig. 1:**
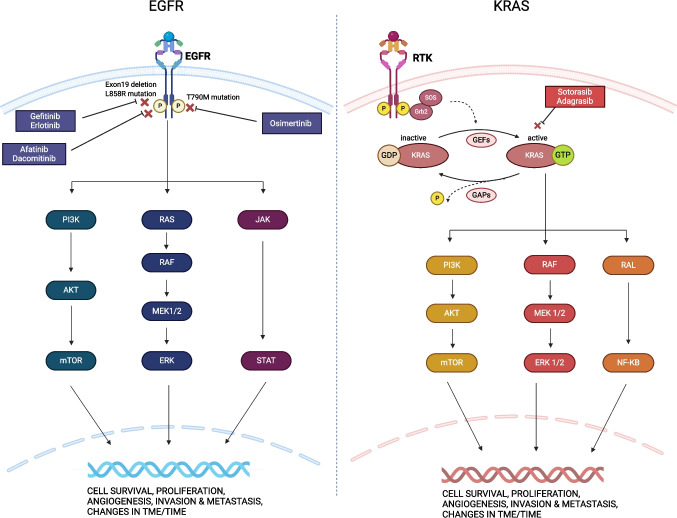
Schematic representation of EGFR and KRAS signaling pathways and associated targeted therapies. The figure illustrates molecular cascades initiated by the activation of the two oncogenes, highlighting their pivotal roles in cell proliferation, survival, and tumor promotion. Clinically approved inhibitors targeting EGFR and KRAS G12 C are also depicted

EGFR is a transmembrane receptor tyrosine kinase (RTK) with an extracellular epidermal growth factor (EGF) binding domain, a transmembrane domain, and an intracellular signaling region. It modulates pivotal cellular processes such as proliferation, growth, and inhibition of apoptosis through pathways such as the MAPK, PI3 K/AKT, and JAK/STAT (Fig. 1) [3]. Its oncogenic potential is primarily realized through mutations, such as exon 19 deletions or the L858R missense mutation in exon 21, both affecting the RTK domain. These alterations result in sustained activation independent of ligand presence [4]. While other mutations in exons 18–21 and duplications of exons spanning 18–26 are possible, their occurrence is relatively rare. Uncommon sensitizing *EGFR* mutations, such as G719X, S768I, and L861Q, occur in approximately 10–15% of *EGFR*-mutant cases [5]. In general, *EGFR* mutations are more prevalent in Asians compared to the Western population, women, and never-smoker patients [6].

Tyrosine kinase inhibitors (TKIs) have emerged as targeted therapies against NSCLCs harboring these mutations, including the first-generation drugs gefitinib, erlotinib, and icotinib, the latter of which is approved only in China. These TKIs reversibly bind to EGFR and restrain the binding of ATP to the tyrosine kinase domain. From the second-generation TKIs, afatinib and dacomitinib are irreversible inhibitors of ATP binding that can prolong survival but are also associated with more toxicities. In addition, resistance to these drugs inevitably develops, often driven by the T790M escape mutation [7]. Third-generation TKI osimertinib selectively inhibits EGFR T790M and was approved as a second-line treatment after a phase III clinical study, which demonstrated improved survival compared to chemotherapy in patients with prior failed TKI treatment, along with a more favorable safety profile [8]. Later, osimertinib was shown to prolong progression-free survival (PFS) and cause fewer adverse events (AEs) compared to first-generation TKIs in previously untreated patients with exon 19 deletions or L858R mutations, leading to its approval as a first-line treatment [9]. Despite initial clinical efficacy, patients may experience progression on osimertinib due to factors such as loss of the T790M mutation, acquisition of the C797S mutation, mutations in other oncogenes such as *KRAS*, gene fusions, *MET* amplification, or transformation of the tumor to small-cell lung cancer [10].

Uncommon *EGFR* mutations generally show poorer responses to first-generation EGFR-TKIs (e.g. gefitinib, erlotinib) than common EGFR mutations. Clinical studies have reported that patients with uncommon *EGFR* mutations have significantly shorter PFS, overall survival (OS) and lower overall response rates (ORRs) compared to those with common mutations [11, 12]. Among second-generation EGFR TKIs, afatinib has resulted in higher ORR and longer median PFS compared to gefitinib or erlotinib in this patient subgroup [13].

From the third-generation TKIs, osimertinib has demonstrated potential efficacy in treating certain uncommon *EGFR* mutations. Two phase II trials reported similar ORRs (50–55%) and median PFS ranging from 8.2 to 9.4 months. However, the duration of response (DoR) varied significantly between the studies (11.2 to 22.7 months). Subtype analysis from both trials showed that tumors harboring the L861Q mutation had the longest median PFS, whereas G719X mutations were associated with the shortest PFS [14, 15]. Taken together, EGFR TKIs remain a viable treatment option for NSCLC patients with uncommon EGFR mutations, though the efficacy varies significantly by specific mutation.

Unfortunately, immune checkpoint inhibitors (ICIs) targeting PD-1/PD-L1, including nivolumab, pembrolizumab, and atezolizumab, have failed to demonstrate clinical benefits in EGFR*-*mutated patients [16].

### KRAS in lung cancer

KRAS, a GTPase that functions downstream of the EGFR signaling pathway, also regulates cell growth, proliferation, and survival through signaling cascades involving the MAPK and PI3 K/AKT (Fig. 1). Early work identified its oncogenic potential, primarily attributed to G12 missense mutations at the GTP binding site, hindering GTP hydrolysis and maintaining it in a constitutively active conformation [16]. Other, albeit less frequent, mutations involve amino acids G13 and Q61 [17]. In contrast to *EGFR*, *KRAS* mutations strongly correlate with tobacco smoking and less commonly with Asian inheritance. Moreover, *KRAS* mutations are frequently found in NSCLC adenocarcinomas but rarely in squamous cell carcinomas [18]. Potentially due to the smoking history, *KRAS*-mutant tumors typically manifest a higher tumor mutational burden (TMB) than their *EGFR*-mutant counterparts [19]. Among *KRAS*-mutant subgroups, tumors with the G12D mutation have been associated with the lowest TMB whereas those with G12 C or G13 mutations tend to exhibit higher TMB [20–22].

KRAS was long considered an undruggable target [23]. Given the mutual exclusivity, TKIs against EGFR are ineffective in *KRAS*-mutant NSCLC [24]. However, recent research has led to the development of two clinically relevant mutant-selective KRAS inhibitors, sotorasib and adagrasib. Sotorasib was the first clinically approved KRAS G12 C inhibitor, which was shown to prolong PFS in previously treated G12 C-mutated lung cancers [25]. In late 2022, the second G12 C inhibitor, adagrasib, was approved by the FDA [26]. More targeted therapies, like G12D and pan-KRAS inhibitors, are currently under development.

Regarding antitumor immunity, sotorasib has been shown to induce a pro-inflammatory TME/TIME in immunocompetent mice, and it was suggested to enhance the efficacy of both chemo- and immunotherapies [27]. In general, *KRAS* mutations, particularly G12 C, are associated with high TMB, elevated PD-L1 expression, and a history of smoking [28]. Compared to *EGFR*-mutant tumors, *KRAS*-mutant tumors (particularly those with high TMB or *TP53* co-mutations) demonstrate a more favorable response to currently approved immune checkpoint inhibitors [29].

### Other driver mutations in NSCLC

In addition to *EGFR* and *KRAS*, several other oncogenic driver mutations have been identified in NSCLC. These include alterations in the anaplastic lymphoma kinase *(ALK),* MET proto-oncogene, receptor tyrosine kinase *(MET),* human epidermal growth factor receptor 2 *(HER2*), and ROS1 proto-oncogene receptor tyrosine kinase *(ROS1)* and account to 9–16%of NSCLC cases*.* While numerous other driver mutations can also contribute to NSCLC pathogenesis, this review will focus on these four, which represent key molecular subsets with available or emerging targeted therapies.

Alterations *in ALK, MET, HER2, and ROS1* lead to the activation of key downstream oncogenic signaling pathways such as MAPK, PI3 K/AKT, and JAK/STAT [30–33]. These alterations are targetable and several TKIs have been developed. For instance, the second generation ALK-TKI alectinib has demonstrated superior efficacy and a more favorable toxicity profile compared to first-generation crizotinib, while the third-generation TKI lorlatinib significantly prolonged PFS and reduced the risk of central nervous system progression compared to crizotinib [30, 34]. Moreover, patients with *MET* exon 14 skipping mutations showed clinical benefit in response to the selective TKIs capmatinib and tepotinib in two phase II studies [35, 36]. Although responses to HER2-targeted TKIs, such as afatinib or dacomitinib, has ranged from minimal to modest, certain HER2 mutation subtypes may show better response to these agents than others [37, 38]. Finally, In the case of rare ROS1 rearrangements, crizotinib, initially developed as a MET/ALK inhibitor, has shown high efficacy in both preclinical and clinical studies [39, 40]. Other multikinase TKIs, such as entrectinib and ceritinib, have also demonstrated clinical activity in *ROS1*-mutated setting [41].

## Immunotherapies in lung cancer

### The advent of immunotherapies

In the past decade, immunotherapies have revolutionized cancer care in selected cancer types. Since the CTLA-4 inhibitor ipilimumab showed unprecedented efficacy in treating unresectable melanoma, immunotherapy development has heavily focused on ICIs. ICIs have been applied in various cancer types, including lung cancer, and are being investigated as single agents and combination therapies. PD-1/PD-L1 blockade has emerged as the most promising avenue in treating NSCLC. Currently, several molecules are approved for clinical use: the anti-PD-1 antibodies nivolumab, pembrolizumab, and cemiplimab, as well as the anti-PD-L1 molecules atezolizumab and durvalumab. However, only a subset of patients benefit from the current ICIs, and the detailed mechanisms of response and resistance remain to be elucidated [42].

Although PD-L1 expression in tumor cells guides the use of anti-PD-1/PD-L1 in NSCLC and other cancers, its role as a biomarker is controversial due to its dynamic nature and limited predictive accuracy [43]. A meta-analysis of 45 studies, across 15 tumor types revealed that PD-L1 expression was predictive only in 28.9% of cases [44]. Another study noted significant variation in the methods used to assess PD-L1 expression across clinical studies, raising concerns about their comparability [45]. Even so, immunotherapy treatments have shown promise, particularly in patients with high expression of PD-L1 [46]. Beyond PD-1/PD-L1, thousands of new ICI drugs and combinations are under development. Drugs targeting checkpoints like LAG-3 and TIGIT, for example, are drawing considerable interest in lung cancer for their potential to enhance immune responses, either as single agents and/or in combination [47, 48]. Below, the most important findings concerning ICIs in the *EGFR*- and *KRAS*-mutated NSCLCs (Table 1), and other tumors, are summarized.

**Table 1:** Clinical trials including immunotherapies in *EGFR*- or *KRAS*-mutant lung cancer

EGFR-related				
study	Identifier	Phase	Drug	Outcome
TATTON	NCT02143466	Ib	Durvalumab with osimertinib	Discontinued due to toxicity [52]
KEYNOTE-010	NCT01905657	II/III	Pembrolizumab vs. docetaxel	No OS benefit for *EGFR-*mutant [50]
KEYNOTE-021	NCT02039674	I/II	Pembrolizumab with erlotinib or gefitinib	Pembrolizumab + gefitinib: discontinued due to toxicity, pembrolizumab + erlotinib no ORR improvement [53]
CheckMate-057	NCT01673867	III	Pembrolizumab vs. docetaxel	No PFS or OS benefit for *EGFR*-mutant [51]
IMpower150	NCT02366143	III	Atezolizumab (A) with bevacizumab (B) and carboplatin/pemetrexed (CP)	OS benefit in ABCP vs. BCP [74]
ORIENT-31	NCT03802240	III	Sintilimab with bevacizumab biosimilar and chemotherapy	PFS benefit of combination therapy [62]
CAURAL	NCT02454933	III	Durvalumab with osimertinib	Discontinued because of toxicity in TATTON study [75]
PACIFIC	NCT02125461	III	Durvalumab after chemoradiation	No OS benefit in *EGFR*-mutant (46.8 months vs. 43 months placebo) [55]
IMPower010	NCT02486718	III	Adjuvant atezolizumab	Potential benefit, but small subgroup size [57]
KEYNOTE-091	NCT02504372	III	Adjuvant pembrolizumab	Better effect in *EGFR*-mutant vs. *EGFR*-wt, but limited subgroup size
				(HR = 0.44 vs. 0.78) [58]
KRAS-related study	**Identifier**	**Phase**	**Drug**	**Outcome**
KRYSTAL-7	NCT04613596	II/III	Pembrolizumab with adagrasib	Currently ongoing, preliminary ORR benefit in PD-L1 ≥ 50%
KEYNOTE-189	NCT02578680	III	Pembrolizumab with chemotherapy	Pembrolizumab plus chemotherapy effective regardless of *KRAS* status [76]
CheckMate-057	NCT01673867	III	Nivolumab vs. docetaxel	Nivolumab more favorable in *KRAS*-mutant [51]
CodeBreaK 101, CodeBreaK 202	NCT04185883, NCT05920356	Ib/II, III	Sotorasib in various combinations (incl. pembrolizumab, atezolizumab)	Currently ongoing
SUNRAY-01	NCT06119581	III	LY353798 (KRAS G12 C inhibitor) with pembrolizumab	Currently ongoing
TRITON	NCT06008093	III	Tremelimumab (anti-CTLA4) with chemotherapy vs. pembrolizumab with chemotherapy	Currently ongoing

### *EGFR* mutations and ICI response

Initially, EGFR activation was reported to upregulate PD-L1 suggesting that ICIs might benefit these patients. However, subsequent studies revealed that *EGFR*-mutant patients typically do not benefit from ICIs [49]. For instance, a phase II clinical study found pembrolizumab ineffective in TKI-naïve patients with advanced NSCLC and high PD-L1 expression (≥ 50%) in the *EGFR*-mutant setting [50]. Similarly, a phase III study showed nivolumab did not improve OS over docetaxel in patients with *EGFR*-mutant NSCLC [51]. The notion that *EGFR* mutations are correlated with high PD-L1 expression has since been challenged, and it has been suggested that *EGFR* mutations are associated with low PD-L1 and low tumor immunogenicity, possibly explaining the clinical outcomes [49].

Moreover, combining ICIs with TKIs has shown significant toxicity. For instance, the concurrent administration of osimertinib and durvalumab resulted in interstitial lung disease-related AEs in over a third of patients [52]. The KEYNOTE-021 phase I/II study combining pembrolizumab and gefitinib reported hepatic impairment in 71.4% of patients, leading to treatment discontinuation [53]. Lastly, recent findings indicated a high incidence of immune-related AEs with durvalumab when used concurrently with chemotherapy or prior to osimertinib treatment [54].

Importantly, the association between ICI efficacy and driver mutation status is not limited to advanced disease. For example, the PACIFIC trial demonstrated that durvalumab significantly improved PFS and OS when used after chemoradiation in stage III NSCLC, however, retrospective analyses suggest that this benefit was restricted to *EGFR* wild-type tumors [55]. Supporting this, a recent multicenter retrospective study compared consolidation osimertinib, durvalumab, and observation in patients with unresectable stage III *EGFR*-mutant NSCLC treated with chemoradiation. Patients who received consolidation osimertinib had significantly longer 24-month real-world PFS (86%) than those receiving durvalumab (30%) or no consolidation (27%), with no unexpected safety signals observed [56].

Interestingly, in IMpower010, a subgroup analysis suggested that patients with PD-L1-positive *EGFR*-mutant tumors could benefit from adjuvant atezolizumab, though this finding was based on a small sample size [57]. The KEYNOTE-091 trial also included *EGFR*-mutant patients, and although the hazard ratio (HR) was better for *EGFR*-mutant patients (0.44 vs. 0.78), the size of the subgroup was fairly limited. Later, in the perioperative KEYNOTE-671 trial, *EGFR* mutation testing was not included, limiting any insights for neoadjuvant setting [58].

Specific *EGFR* mutations may influence ICI responses. A retrospective study revealed that not all *EGFR* mutations have a similarly grim prognosis for ICI efficacy: while exon 19 mutations associated with poorer ICI responses, L858R mutations showed no difference compared to *EGFR* wild-type cancers and were linked to higher TMB [59]. Another study, investigating the effects of TMB on response to TKIs in *EGFR*-mutant NSCLC, confirmed the association of the L858R mutation and a higher TMB [60]. A retrospective study evaluating the effect of ICI monotherapy in advanced NSCLC found marked differences in PFS and OS between different *EGFR*-mutant subgroups, with exon 21-mutated tumors showing more favorable outcomes than those with exon 19 or T790M mutations. The same study also showed that PD-L1 expression of ≥ 1% was associated with increased PFS among *EGFR*-mutant patients [29]. The results of a retrospective study comparing the efficacy of ICIs in patients with uncommon versus common *EGFR* mutations in advanced NSCLC suggested that uncommon *EGFR* mutations (G719X, *n* = 9) were associated with a higher median PFS and ORR than common mutations (mPFS: 2.5 months vs. 1.82 months; ORR: 25% vs. 10.94%), although median OS did not significantly differ between the groups [61]. However, the study had some limitations, including a relatively small cohort size and a lack of subgroup analyses.

While in general the results in EGFR-mutant patients may seem discouraging, new combination treatments are under investigation and could offer clinical benefits: The phase III IMpower150 study demonstrated OS improvement with a combination of atezolizumab, bevacizumab, and chemotherapy in patients with sensitizing EGFR mutations. Additionally, the newly developed anti-PD-1 antibody sintilimab showed efficacy in combination with bevacizumab and chemotherapy in patients who had progressed on TKIs [62].

### *KRAS* mutations and ICI response

While *KRAS* mutations typically indicate poor OS compared to tumors with *EGFR* mutations or those wild-type for both *KRAS* and *EGFR* [63], NSCLCs with *KRAS* mutations have demonstrated a higher ORR to ICIs. This improved response might be linked to the elevated levels of tumor-infiltrating lymphocytes (TILs), TMB, and immunogenicity observed in *KRAS*-mutant NSCLCs [64]. In 2015, CheckMate 057 phase III clinical study showed that nivolumab significantly improved OS over docetaxel in non-squamous NSCLC patients previously treated with chemotherapy, with fewer AEs [51]. In another phase III study, atezolizumab extended OS over docetaxel in *KRAS*-mutant patients who had undergone prior chemotherapy compared to *KRAS* wild-type patients. The same study found that while patients with negative/low PD-L1 expression benefitted from atezolizumab, this effect was particularly strong in patients with high PD-L1 expression [65]. Furthermore, a retrospective study of 282 ICI-treated *KRAS-*mutant advanced NSCLC patients revealed significantly better ICI efficacy in patients with higher PD-L1, pointing out PD-L1 expression as a prognostic factor for ICI response in *KRAS*-mutant tumors [66].

Different *KRAS* mutations can also impact cellular signaling and treatment responses [67]. KRAS G12D has been associated with lower PD-L1 expression levels compared to other KRAS mutation subtypes [22]. In contrast, KRAS G12 C has been reported to have higher PD-L1 expression than both other KRAS subgroups and KRAS wild-type tumors [20, 21]. The efficacy of ICI monotherapy in KRAS G12D-mutated NSCLC has been relatively poor. Patients carrying this mutation have demonstrated lower ORR, shorter median PFS, and shorter OS compared to patients with other KRAS subtypes or KRAS G12 C mutations [22, 68]. Interestingly, among KRAS G12D patients, those with no or light smoking history had particularly poor responses to ICI monotherapy compared to heavy smokers, suggesting that TMB or neoantigen load might play a role [22]. In contrast, KRAS G12 C-mutated NSCLC has been associated with better responses to ICI monotherapy relative to other KRAS subtypes, including longer median PFS and higher ORR [69, 70].

The data related to KRAS mutational subtypes and ICI efficacy is still maturing. The ICI response in KRAS-mutant tumors is further complicated by various co-mutations, most notably in *STK11* (LKB1), *TP53*, and *CDKN2 A/B*. Tumors with *KRAS/TP53* co-mutations show a higher number of somatic mutations, elevated inflammatory markers, immune checkpoint molecules, and a better relapse-free survival (RFS). Conversely, *KRAS/STK11* co-mutated tumors exhibit lower PD-L1 levels [71]. *STK11* mutations have been linked to PD-L1 negativity and resistance to PD-1/PD-L1 blockade [72]. Additionally, loss of *STK11* is associated with reduced expression of the stimulator of interferon genes (STING), aiding cancer cell survival and immune evasion [73].

### Other driver mutations and ICI response

The knowledge of clinical efficacy of ICIs for patients carrying *ALK, MET, ERBB2*, and *ROS1* mutations is still limited. This is largely due to small patient cohorts, the pooling of disparate mutations in analyses, and the retrospective nature of many available studies.

A retrospective study involving 23 patients with advanced NSCLC and *ALK* rearrangements treated with ICI monotherapy reported a poor response: the median PFS was approximately 3 months, and nearly 70% of patients experienced disease progression after ICI monotherapy [29]. Similarly, another retrospective study of seven patients with *ALK*-mutated NSCLC reported a median PFS of only 0.6 months with either nivolumab or pembrolizumab. Notably, PD-L1 expression did not correlate with clinical outcomes [77]. In patients with *EGFR* and *ALK* alterations who had developed resistance to TKIs, ICI monotherapy outcomes remained poor [78]. A phase I/II study combining nivolumab and crizotinib in *ALK*-rearranged NSCLC was terminated early due to hepatotoxicity [55]. Overall, limited evidence exists for the use of ICI-based combination therapies in *ALK*-mutated NSCLC, largely because these patients are frequently excluded from clinical trials [79, 80].

The efficacy of ICI monotherapy in NSCLC with *MET* exon 14 skipping mutations is controversial. While two retrospective studies reported low median PFS (ranging from 1.9 to 4.9 months), another reported a median PFS of 24.7 months—substantially longer than previously observed. ORRs also varied, ranging from 17% to 42.9%. Combination therapy may improve outcomes in these patients: in a retrospective study patients receiving ICI + chemotherapy had a significantly longer median PFS (by 3.5 months) and median OS (by 4 months) compared to those treated with chemotherapy alone [81].

In HER2-mutated advanced NSCLC, most evidence suggests limited benefit from ICI monotherapy. A retrospective study reported disease progression after ICI monotherapy in 67% of patients, with a median OS of ~ 20 months, independent of PD-L1 expression levels [29]. Another study reported a similarly short PFS of 2 months [82]. On the other hand, a retrospective study evaluating ICI ± chemotherapy combinations in HER2-positive NSCLC patients observed a 52% ORR in the ICI + platinum-doublet group, with a 1-year median PFS of 6 months—higher than prior reports. In contrast, patients receiving ICI monotherapy had an objective response rate of only 16% and a median PFS of 4 months [83]. Additionally, a small study from the Chinese Lung Cancer Center, involving five patients with HER2 insertions or amplifications, reported a median PFS of 8 months with ICI + chemotherapy [84]. These findings suggest that combination strategies may hold promise in this subset of NSCLC.

For *ROS1*-rearranged NSCLC, responses to ICI monotherapy have also been modest. A retrospective study of seven patients reported a 17% ORR and disease progression within two months in approximately 43% of cases [29]. However, a later study of patients treated with first-line pembrolizumab combined with chemotherapy showed more promising results: the median PFS exceeded 24 months, and the ORR was 83%. In contrast, patients receiving later-line ICI + chemotherapy combinations had a median PFS of only 5.8 months and an ORR of ~ 29% [85].

## *EGFR* and *KRAS* mutations differentially impact the TIME

### Mutations affect the tumor immune microenvironment

**Fig. 2:**
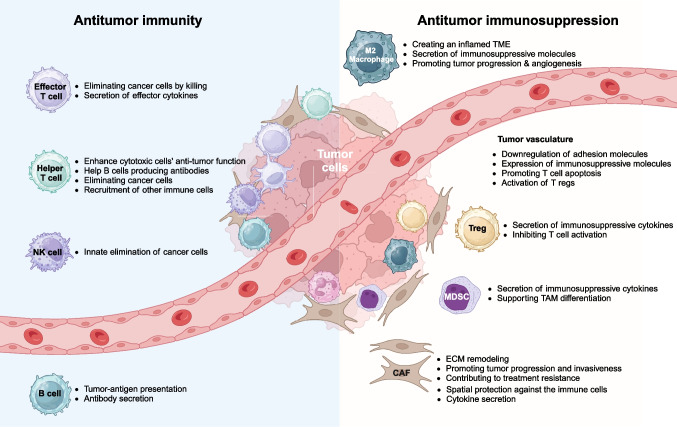
Cellular components of the malignant TME/TIME. The main cell types with antitumor or immunosuppressive functions within the TME/TIME are illustrated and the mechanisms behind antitumor or tumor-supporting activity by each cell type are highlighted. Effector T cell: cytotoxic CD8^+^ T cell; Treg, regulatory T cell; Helper T cell: CD4^+^ T cell; MDSC: myeloid-derived suppressor cell; CAFs: cancer associated fibroblasts

Genetic mutations play a significant role in modulating the TME/TIME ultimately facilitating immune evasion and tumor progression [86]. However, the genotype of a cancer cell alone isn't enough to fully explain how it orchestrates the TME/TIME, predicts tumor behavior, or determines clinical outcomes. Oncogenic alterations, such as mutations in *EGFR* and *KRAS*, along with their downstream signaling pathways, give unique characteristics to cancer cells that shape the TME/TIME. Furthermore, the interplay among tumor cells, immune cells, stromal components, and endothelial cells results in a heterogeneous microenvironment characterized by varying degrees of inflammation and hypoxia—both of which are critical drivers of tumor progression and clinical prognosis (Fig. 2).

The composition of the TME/TIME in NSCLC is indicative of the cancer’s stage and has prognostic significance, with ongoing research focused on targeting these components for therapy [87]. Yet, the differences in the TME/TIME between *EGFR-* and *KRAS*-mutant tumors, as well as their mutational subtypes, underscore the challenge of developing a one-size-fits-all immunotherapy approach for the genetic subtypes of NSCLC.

### Tumor-infiltrating lymphocytes

**Fig. 3:**
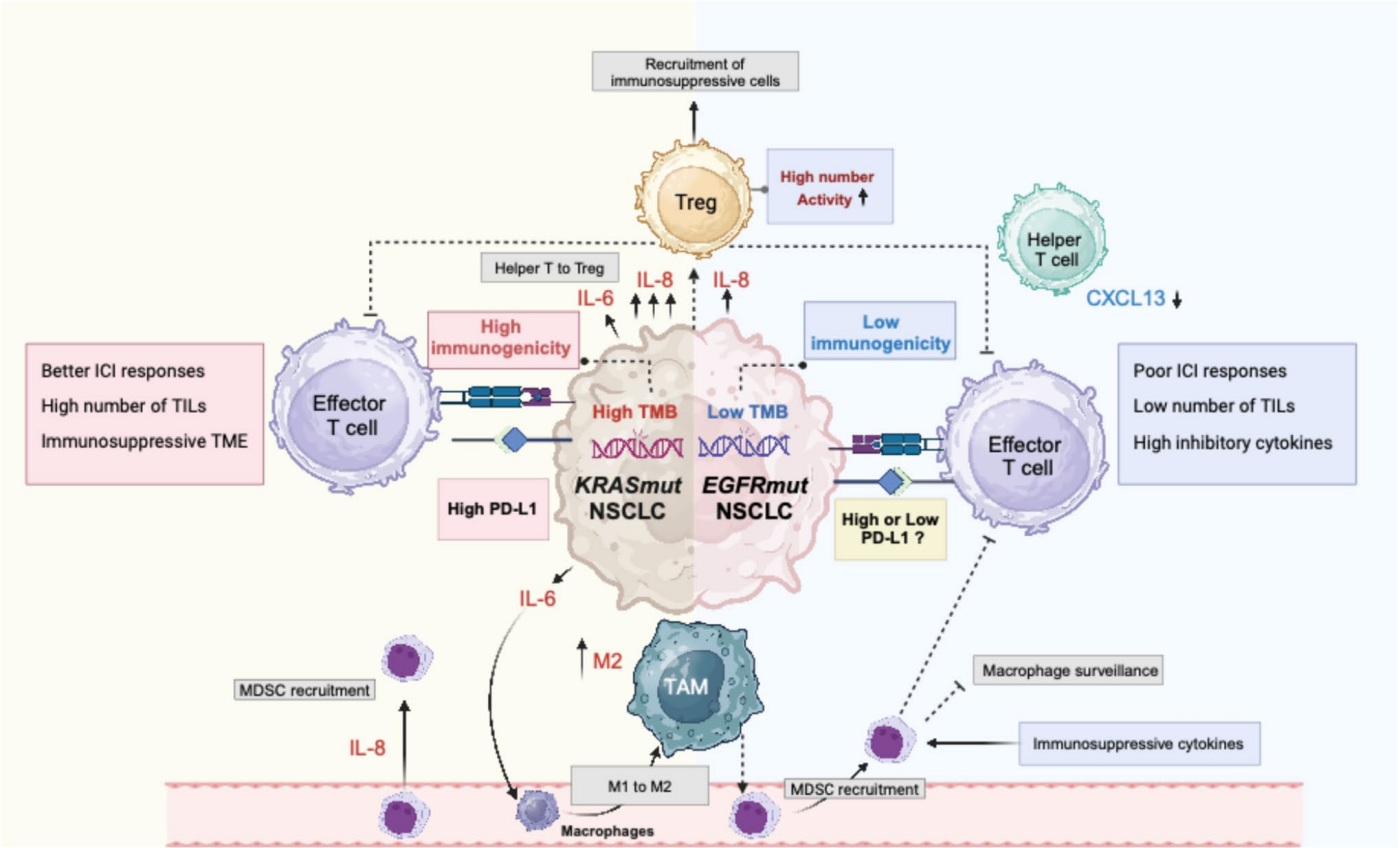
The TME/TIME in EGFR-mutated versus KRAS-mutated NSCLC. The oncogenic mutations in EGFR and KRAS orchestrate their TME/TIME by different mechanisms. By secreting cytokines, chemokines, growth factors or up/down-regulation of different receptors, tumor cells hijack different components of the TME/TIME to promote their growth and progression

TILs consist of all lymphocytes present in a tumor and generally encompass T cells, B cells, and natural killer (NK) cells. Among these cells are regulatory T cells (Tregs), which maintain immune homeostasis, but can also inhibit immune cell-mediated tumor killing and induce the formation of an immunosuppressive microenvironment [88]. *EGFR* and *KRAS* mutations in tumor cells can modulate the function of TILs through complex signaling networks, including secretion of chemokines and cytokines present in the TME/TIME (Fig. 3).

### Cytotoxic, helper, regulatory T cells, and cytokines

Due to their role as effectors in antitumor immunity and immunotherapy responses, cytotoxic (CD8^+^) T cells have been extensively investigated. High cytotoxic T cell infiltration generally correlates with increased OS in lung cancer, whereas cytotoxic TILs expressing immune inhibitory markers such as PD-1, TIM-3, and LAG-3 are associated with an exhausted immune phenotype and poor prognosis [89, 90].

An early study on murine lung tumors suggested that *EGFR*-mutant tumors are characterized by an immunosuppressive microenvironment involving low cytotoxic and helper TIL amounts, high number of Tregs, and high expression of inhibitory cytokines and immune checkpoints [91]. However, despite the high PD-1/PD-L1 expression previously observed in both mice and patients, clinical studies revealed poor response to PD-1 blockade, indicating that immunosuppressive events beyond PD-1/PD-L1 expression, such as the levels of cytotoxic T cells or other cellular subtypes, might contribute to ICI response [92].

Cancer cells secrete several factors, including cytokines, chemokines, and exosomes, that play an important role in modulating the TME/TIME. The secretion of these factors is regulated by signaling from oncogenes, such as *EGFR* and *KRAS*. Preclinical and clinical studies have shown that TGF-β is upregulated in *EGFR-*mutated lung cancer through the EGFR-ERK1/2-p90RSK signaling pathway, resulting in low CD8 + T cell infiltration, and impaired anti-tumor immunity [93]. Dual blockade of TGF-β and PD-1 resulted in enhanced CD8 + T cell infiltration and anti-tumor activity in mouse models.

Another study using clinical specimens showed higher Treg infiltration, less CD8 + T cells, lower TMB and lower PD-L1 expression in *EGFR*-mutant tumors compared to *EGFR*-wild type tumors [94]. Furthermore, EGFR signaling was shown to lower expression of cytokines such as CXCL10 and CCL5, both essential for T cell recruitment. The cytokine signatures were reversed by erlotinib. In addition, CCL22, which helps to recruit Tregs, was elevated with activation of EGFR signaling and reversed by erlotinib.

EGFR signaling also influences the immune landscape by modulating MHC expression. Both EGFR TKIs and antibodies, such as cetuximab, were shown to enhance the induction of MHC class I and II molecules in human keratinocytes [95]. Notably, MHC class I protein levels increased even in the absence of IFN-γ. Clinical biopsy samples further supported these findings, showing elevated epidermal MHC I and II expression during EGFR inhibitor therapy. These results suggest that EGFR inhibition may restore antigen presentation capacity, thereby improving immune visibility of tumor cells.

On the other hand, oncogenic *KRAS* signaling has been shown to induce the secretion of several cytokines that contribute to inflammation and immune evasion. IL-8 is a direct transcriptional target of KRAS signaling and promotes angiogenesis, inflammation, and tumor progression [96]. Among the CXCR2 ligands, CXCL3 is highly secreted in *KRAS*-mutant preclinical models and contributes to immunosuppressive myeloid cell recruitment [97]. CXCR2 ligands such as CXCL1, CXCL2, CXCL3, and CXCL5 are highly expressed in the tumor microenvironment of *KRAS*-mutant preclinical models [97–99], although their elevated secretion is often attributed to stromal or immune cells rather than direct production by *KRAS*-mutant tumor cells. Oncogenic *KRAS* signaling via the ERK–MAPK–AP-1 pathway induces the expression of immunosuppressive cytokines such as TGF-β1 and IL-10, as shown in *KRAS*-mutant tumor models [100]. High PD-L1 expression is frequently observed in *KRAS*-mutant tumors, and one study demonstrated that this upregulation is driven by ERK signaling, contributing to CD3⁺ T cell apoptosis, which can be reversed by dual inhibition of p-ERK and PD-1/PD-L1 [101]. Additionally, PD-L1 expression is stabilized at the mRNA level via MEK-mediated phosphorylation of tristetraprolin (TTP), an RNA-binding protein that normally promotes PD-L1 mRNA degradation [102]. The role of KRAS in shaping the TIME was further demonstrated by *in vivo* imaging mass cytometry following KRAS-G12 C inhibition, which enhanced CD8⁺ T cell proliferation, cytotoxic function, and PD-1 expression, as well as CXCL9 production by dendritic cells [103].

A multi-omics study on patient samples consistently confirmed that *KRAS* mutations, especially when co-occurring with mutant *TP53,* correlated with response to PD-1 blockade, increased PD-L1 expression, and higher T cell infiltration [104]. When comparing *KRAS* mutation subtypes, Ricciuti *et al*. showed that KRAS G12D tumors had significantly fewer cytotoxic T cells at the tumor-stroma interface compared to non-G12D subtypes. Additionally, the proportions of intratumoral PD-1^+^ T cells and cytotoxic PD-1^+^ T cells were also lower in G12D tumors [22]. Consistently, Liu et al. reported that cytotoxic T cell infiltration was lower in KRAS G12D tumors, and that these tumors more frequently lacked TILs altogether, suggesting that the TIME in KRAS G12D may be poorly immunogenic or unable to recognize tumor-associated antigens [68].

Helper (CD4^+^) T cells are essential immune system regulators assisting other immune cells to function. Generally, a high frequency of helper TILs in lung tumors correlates with higher OS [90]. Unfortunately, little research has been conducted on the composition of helper T cells in the EGFR-mutant NSCLC. One study suggested that follicular helper T cells producing the B cell attracting molecule CXCL13, as well as tissue resident memory (TRM) CD8^+^ TILs, are depleted in EGFR-mutant tumors, further contributing to immunosuppression [105]. However, no differences in helper TIL amounts could be observed between responders and non-responders to nivolumab in EGFR T790M + NSCLC patients, even though PFS was short in all cohorts of this study [106]. In the context of KRAS, it was shown that in the early phase of NSCLC, TILs express PD-1, and that PD-1 blockade in a KRAS-mutant murine NSCLC model increased both T cell proliferation and helper T cell activity. The presence of helper T cells was crucial for treatment efficacy, highlighting that cytotoxic T cell functions alone are insufficient for successful ICI treatment [107].

In colon cancer, KRAS induced the conversion of helper T cells into Tregs [100], a finding later confirmed in pancreatic cancer [108]. In lung cancer, KRAS-mutant tumors were found to upregulate Tregs through IL-6 signaling [109]. Consistent with this, IL-6 signaling was associated with impaired cytotoxic T cell function and resistance to atezolizumab [110]. Besides IL-6, both *KRAS*- and *EGFR*-mutant NSCLCs have been shown to overexpress IL-8, but the effect was more prominent in the KRAS-mutant tumors. Interestingly, IL-8 promotes cancer cell growth and migration, and is secreted by Tregs as a chemoattractant to recruit neutrophils and myeloid-derived suppressor cells (MDSCs) to the tumors. These MDSCs, in turn, contribute to an immunosuppressive TME/TIME through various pathways, including inhibition of T cell activation, secretion of suppressive factors, and cytokine-mediated activation of Tregs, creating an immunosuppressive feedback loop [111, 112].

### B cells

Despite not attracting the same interest as T cells, more recently, B cells have emerged as significant contributors to the NSCLC microenvironment. Bruno *et al.* showed that B cells can present tumor antigens to helper T cells residing in the tumor tissue, but can also become exhausted, and the exhausted state of B cells is associated with increased Treg frequency [113]. However, the association between EGFR and KRAS mutations on B cell status in the TME/TIME is not well known. One *in silico* study linked KRAS mutations to lower infiltration of B cells in lung adenocarcinoma, whereas another study correlated EGFR mutant tumors with low B cell amounts [92]. However, comprehensive *in vitro* or *in vivo* studies investigating possible links between mutational status and tumor B cells are yet to be conducted.

### NK cells

NK cells are innate immune cells that play a critical role in identifying and eliminating cancerous cells through their cytotoxic abilities and cytokine secretion. It has been suggested that NK cells in lung cancer have lower cytotoxicity than the ones found in the bloodstream, potentially due to the presence of immunosuppressive alveolar macrophages in the lungs [114]. The ineffectiveness of infiltrating NK cells in NSCLC is thought to be a product of the immunosuppressive TME/TIME, in which NK cells are exhausted by Tregs, MDSCs, and immunosuppressive cytokines [115]. Cong *et al*. showed that NK cells could inhibit tumor initiation, but not progression, in *KRAS*-mutant murine lung tumors and that their number decreased as the disease progressed. While the potential benefits of reversing NK exhaustion, as well as NK infusion therapies have shown some promising results, decisive studies on NK cells in *EGFR*- and *KRAS*-mutant tumors are still missing [116]. One study found that injection of autologous NK cells could increase PFS in EGFR-mutant patients [117], but similar studies with KRAS-mutant tumors are lacking. It has also been found that treatment with TKIs erlotinib and gefitinib rendered lung cancer cell lines susceptible to NK-mediated killing [118], suggesting possible synergistic effects of targeted treatments and upcoming NK cell therapies.

### Macrophages

Tumor-associated macrophages (TAMs) can be crudely categorized as either pro-inflammatory/anti-tumorigenic (type M1) or anti-inflammatory/pro-cancerous (type M2), albeit this view might be deemed simplistic. However, the high number of M2 TAMs has been linked to poor prognosis in NSCLC [119]. Moreover, the abundance of M2 TAMs is related to high expression of PD-L1 and poor survival [120].

Higher levels of M2 macrophages were found in EGFR wild-type than EGFR-mutant patients, and in smokers than non-smokers [121], suggesting a possible correlation with mutant KRAS. Consistently, KRAS mutations were associated with a higher number of TAMs and macrophage reprogramming via tumor-derived lactate and colony stimulating factor in colorectal cancer [122]. In pancreatic cancer, contact with KRAS-mutant cells led to higher expression of M2 markers and increased pro-tumor effects of macrophages [123]. In a lung cancer mouse model, secretion of IL-6 by KRAS-mutant tumor cells correlated with increased numbers of M2 TAMs and tumor growth [109]. In chemotherapy resistant lung cancer, presence of M2 macrophages correlated with KRAS mutation and poor survival [124]. In the EGFR-mutant context, single-cell RNA sequencing data suggested that macrophages within EGFR-mutant tumors upregulate the expression of immunosuppressive cytokines, leading to MDSC recruitment and macrophage surveillance suppression, as well as inhibition of cytotoxic T cell activation by dendritic cells [125]. EGFR-mutant group also exhibited less active cytotoxic T cells. The further impact of EGFR or KRAS mutations on the role of TAMs in an immunotherapy setting remains to be investigated.

### Cancer-associated fibroblasts

Cancer-associated fibroblasts (CAFs) possess many tumorigenic properties, including the support of tumor growth and metastasis through epithelial-to-mesenchymal transition (EMT), metabolic reprogramming, and immunosuppression. The presence of CAFs is associated with treatment resistance across a wide variety of cancer types and treatments. However, CAFs constitute a heterogeneous population, and only recently, specific CAF subtypes and their impact on prognosis have been studied in detail at the single cell level. For example, specific NSCLC myofibroblast populations were associated with poor OS rates and altered immune composition including increased number of macrophages and neutrophils [126]. An integrative single-cell study on fibroblasts originating from different tissues revealed that *LRRC15* + alveolar fibroblasts and *NPNT* + myofibroblasts were enriched in NSCLC tumors compared to other tumor types [127]. Another study discovered that specific subpopulations of CAFs emerge as lung cancer progresses, conferring spatial protection against T cell infiltration [128]. Imaging mass cytometry analysis of NSCLC CAFs revealed that specific CAF subpopulations are associated with immune infiltration patterns and NSCLC prognosis, and their spatial distribution was also highly variable [129].

CAFs and their subtypes also have a significant impact on therapy responses, including responses to EGFR inhibitors. For example, CAFs have been shown to confer resistance to EGFR TKIs and sole presence of CAFs from gefitinib-resistant tumors can induce EMT and resistance to TKIs in previously sensitive cancer cells [130, 131]. They can modulate the recruitment and activity of various immune cell types, including Tregs and dendritic cells, through the secretion soluble molecules like TGF-β and VEGF. CAFs can also play a critical role in regulating the TIME to affect ICI responses [132].

### Tumor vasculature

Angiogenesis has been long recognized as a cancer hallmark. Tumor endothelial cells (TECs) contribute to immune evasion through downregulation of adhesion molecules, expression of immunosuppressive molecules, by promoting T cell apoptosis, and by activating Tregs [133]. The interplay between EGFR- and KRAS-mutant tumors and angiogenesis is reasonably well established: EGFR mutations have been linked to increased VEGF secretion in NSCLC cell lines, and multiple studies have found that KRAS contributes to the upregulation of VEGF and IL-8 in various cancers, essential for tumor angiogenesis [134]. Although EGFR mutations are known to promote angiogenesis by upregulating VEGF expression via STAT3 activation, neither EGFR nor KRAS mutations were significantly associated with VEGF-A expression in a clinical association study [135]. Regardless, in the phase III Impower150 study, patients with EGFR mutations benefitted from atezolizumab in combination with chemotherapy and anti-VEGF bevacizumab, suggesting that endothelial cells are critically involved in these tumors [136]. Similarly, a phase III trial in NSCLC patients with EGFR mutations revealed improved PFS for the combination therapy of bevacizumab and erlotinib versus erlotinib alone, though with a higher incidence of AEs [137]. However, tumors with KRAS mutations, particularly those with G12D mutations, experienced inferior PFS and OS when treated with a combination of bevacizumab and chemotherapy. This indicates that despite the connection between KRAS and angiogenesis, bevacizumab may not be an effective treatment option for these patients [138].

## The effect of TKI treatment on the TIME

The significant clinical benefit observed in *EGFR*-mutant NSCLC patients following EGFR TKI therapy suggests that the efficacy of these treatments extends beyond direct tumor cell targeting. An immune-mediated component likely plays a role in the therapeutic response. For instance, skin rash is a commonly reported side effect of TKI treatment, indicating increased circulating lymphocytes and cytokines derived from an inflamed tumor microenvironment [139].

EGFR TKI therapy induces dynamic shifts in immune marker expression that fluctuate across two distinct phases: during active treatment and upon the development of acquired resistance. Clinical studies involving first- and second-line TKIs, including osimertinib, have demonstrated low T cell infiltration in the TME of treatment-naïve patients and those with TKI-acquired resistance [78, 140].

Despite low T cell infiltration in *EGFR*-mutant NSCLC tumors during both active treatment and acquired resistance phases, these tumors exhibit increased expression of immunosuppressive markers. Specifically, there is enrichment of PD-1 expressing T cells, Tregs, and IDO1 expressing macrophages, suggesting that the tumor microenvironment becomes more immunosuppressive over time. In contrast, samples obtained during EGFR TKI treatment reveal a pro-inflammatory tumor microenvironment characterized by increased T cell infiltration, fewer dysfunctional T cells compared to baseline or progressed stages, and increased infiltration of NK/NKT cells [140].

Similarly, EGFR TKI therapy has been shown to enhance immune cell infiltration and cytotoxicity in treatment-responsive EGFR-mutant NSCLC patients. However, these effects are absent following the development of TKI resistance [141]. Specifically, no significant changes in anti-tumor cell infiltration or cytotoxicity were observed between pre-treatment and post-progression samples, indicating a lack of immune remodeling after resistance develops. The therapy also upregulated several genes involved in IFN-γ signaling and immune checkpoint regulation, including *PDCD1* (encoding PD-1) and *BTLA*. This upregulation may contribute to immune evasion mechanisms in the tumor microenvironment.

PD-L1 upregulation has been reported in patients with acquired TKI resistance. In a cohort of 138 *EGFR*-mutant NSCLC patients who underwent re-biopsy after developing TKI resistance, the proportion of patients with PD-L1 expression ≥ 50% increased from 14% at baseline to 28% at the resistance [142]. This finding aligns with previous studies showing increased PD-L1 expression during the resistance phase and its association with poor clinical outcomes [143]. Hsu *et al*. [144] reported that higher PD-L1 expression (≥ 50%) in advanced *EGFR*-mutant NSCLC patients was associated with poorer outcomes in terms of PFS and OS when treated with first-line osimertinib. In contrast, the FLAURA trial demonstrated that PD-L1 expression status did not significantly affect response to first-line osimertinib, with similar PFS in PD-L1-positive and PD-L1-negative groups (18.9 months vs. 18.4 months, respectively) [145]. Therefore, the correlation between PD-L1 expression and TKI sensitivity remains uncertain, highlighting the need for further investigation in larger and more representative patient cohorts.

TMB has been observed to increase upon the development of EGFR TKI resistance compared to baseline [60, 142]. Further analysis using proteomic techniques on plasma samples from 25 *EGFR*-mutant NSCLC patients treated with osimertinib (either first- or second-line) identified several immune-related proteins with altered expression following the onset of TKI resistance [146]. CD27, CD70, CXCL13, FASLG, ICOSLG, and LY9 were significantly downregulated, while NECTIN4 was upregulated at progression. Notably, patients with low NECTIN4 expression demonstrated improved OS, suggesting that NECTIN4 levels could be a valuable prognostic marker for patient outcomes.

Collectively, these findings highlight the immunomodulatory effects of EGFR TKIs and the importance of characterizing immune dynamics across treatment stages. A better understanding of these dynamic changes could inform the development of immune-related biomarkers and guide the design of more efficient therapeutic approaches to improve clinical outcomes. However, several limitations are noted in the studies reported: first, the small sample size and lack of matching samples from different treatment phases; second, the limited availability of treatment or minimal residual disease samples during treatment or post-treatment; third, the fact that a fraction of patients receive chemotherapy between EGFR TKI treatment and re-biopsy; and finally, the need for more studies specifically evaluating osimertinib-induced changes in the TME/TIME as opposed to conventional TKIs.

## Synopsis

Immunotherapies have already proven effective for NSCLC, but they benefit only a subset of patients. With the rapid development of novel immunotherapeutic agents including T cell targeted drugs, cancer vaccines, oncolytic viruses, and cell therapies, understanding the mechanisms of primary and acquired immunotherapy resistance becomes imperative. This also makes it necessary to investigate the role of the TME/TIME in immunotherapy resistance more closely. New methodologies, such as single-cell and spatial approaches, are enabling more detailed investigations on the role of tumor-associated cells, including fibroblasts and immune cells. Additionally, targeting CAFs or angiogenesis together with immunotherapies has emerged as a promising new treatment strategy.

It is now clear, that the tumor cell characteristics, such as the mutational landscape, are impacting and forming the characteristics of the TME/TIME. Numerous questions remain about the roles of various cell types in the TME/TIME of NSCLC tumors with either *EGFR* or *KRAS* mutations, essential for understanding ICI responses across patient subgroups. For example, it is still unclear how big the influence of tumor cell-intrinsic properties vs. environmental factors are. Taken together, broadening our understanding of the networks within the TME/TIME in *EGFR*- versus *KRAS*-mutant NSCLC is an important current objective in research with many questions yet to be resolved.

## Data Availability

No datasets were generated or analysed during the current study.
